# Simulator acceleration and inverse design of fin field-effect transistors using machine learning

**DOI:** 10.1038/s41598-022-05111-3

**Published:** 2022-01-21

**Authors:** Insoo Kim, So Jeong Park, Changwook Jeong, Munbo Shim, Dae Sin Kim, Gyu-Tae Kim, Junhee Seok

**Affiliations:** 1 School of Electrical Engineering, Korea University, Seoul, Korea; 2 Computational Science and Engineering Team, Innovation Center, Samsung Electronics, Samsungjeonja-ro, Hwaseong-si, Gyeonggi-do 18448 South Korea; 3 Present Address: Electronic Components Examination Division, Korean Intellectual Property Office, Daejeon, 35208 Korea

**Keywords:** Mathematics and computing, Nanoscience and technology, Physics

## Abstract

The simulation and design of electronic devices such as transistors is vital for the semiconductor industry. Conventionally, a device is intuitively designed and simulated using model equations, which is a time-consuming and expensive process. However, recent machine learning approaches provide an unprecedented opportunity to improve these tasks by training the underlying relationships between the device design and the specifications derived from the extensively accumulated simulation data. This study implements various machine learning approaches for the simulation acceleration and inverse-design problems of fin field-effect transistors. In comparison to traditional simulators, the proposed neural network model demonstrated almost equivalent results (R^2^ = 0.99) and was more than 122,000 times faster in simulation. Moreover, the proposed inverse-design model successfully generated design parameters that satisfied the desired target specifications with high accuracies (R^2^ = 0.96). Overall, the results demonstrated that the proposed machine learning models aided in achieving efficient solutions for the simulation and design problems pertaining to electronic devices. Thus, the proposed approach can be further extended to more complex devices and other vital processes in the semiconductor industry.

## Introduction

Since the development of metal–oxide–semiconductor field-effect transistor (MOSFET) device, it took us only 70 years to carry a computer in our pockets that is a billion times faster than the first computer. However, the compression of the traditional MOSFET to the nanoscale has induced certain physical limitations such as the short-channel effects. Consequently, new MOSFET devices such as a fin field-effect transistor (FinFET) and a gate-all-around field-effect-transistor device have been proposed to surpass these limitations^1,2^.

The recently suggested MOSFET devices pose new challenges related to their designs. In particular, the establishment of an appropriate design that meets the desired specifications is one of the major design problems regarding these new MOSFET devices, referred to as the inverse-design problem. Thus, a potential solution involves testing numerous devices with various designs until the desired device is determined. However, such a solution is not feasible because the manufacturing of numerous MOSFET devices with distinct designs for reviewing their specifications is a time-consuming and expensive endeavor. Therefore, simulation-based estimation of specifications can be considered as a reasonable approach to review the design of a FinFET device^3^. Nevertheless, the conventional concept of obtaining solutions from differential equations describing physical laws is still a complex task and requires the prior knowledge of an expert. In addition, this approach can predict only the specifications of the devices from the design, typically the device parameter. Thus, these facts imply that the one-way approach is an unsuitable solution for the inverse-design problem.

Recently, the explosive growth of machine learning models such as deep neural networks has provided improved solutions for several complex problems. For instance, deep-generative models have been recently implemented to solve complex inverse-design problems in various fields such as nanophotonics and molecular designs^4–8^. Moreover, recent studies reported that the deep neural networks can provide an efficient solution for solving Maxwell equations, which are partial differential equations calculating the electromagnetic values of a given space^9,10^.

Although the implementation of machine learning and deep learning models have yielded significant advances in several related fields, the direct application of such approaches in the semiconductor design problems has recently been initiated^11^. However, existing studies showed that implementing the deep neural networks might be an useful solution for the semiconductor design problems^12^. In this study, we propose a guideline to implement machine learning models in semiconductor device simulation and design problems. In particular, we applied log-reciprocal normalization for data preprocessing, implemented neural network models with a combined loss function for simulation acceleration, and introduced model-based device design. The experimental results aimed to determine the capability of the proposed machine learning approach in solving both the simulation acceleration and inverse-design problems with adequate accuracy.

## Results and discussion

### Data preprocessing with log reciprocal normalization

The input and output samples used for training and testing the proposed methods were generated using a FinFET simulator constructed with FlexPDE. The FinFET simulator was developed to calculate the device parameter of a given FinFET design. The four design parameters of the FinFET device include the channel top width (${W}_{T}$), channel bottom width (${W}_{B}$), channel thickness (${T}_{Si}$), and backgate voltage (${V}_{Bg}$), which were used as input parameters for the FinFET simulator. In particular, the three device parameters of the FinFET device—the drain current (${I}_{D}$), effective mobility ($\mu$), and electron charge density (${Q}_{N}$)—were regarded as the output values calculated using the simulator. Additionally, three device parameters—subthreshold swing (${S}_{Sw}$), threshold voltage (${V}_{Th}$), and mobility degradation (${\mu }_{Deg}$) of the FinFET device were calculated from the device parameters (${I}_{D}, \mu$) in order to be used as the input values and the evaluation methods of the proposed models. In the paper, the three device parameters directly calculated through the simulators (${I}_{D}$, $\mu ,$ ${Q}_{N}$) were referred as primary properties and three device parameters calculated from the primary properties with the implicit functions were referred as secondary properties. These design and properties are summarized in Table S1. As the original values of the design and primary properties were not in appropriate ranges for training, these values were normalized prior to the training procedure. In particular, the four design parameters and three primary properties were normalized in between 0 and 1 with the min–max normalization and the log-reciprocal normalization, respectively. Moreover, the log-reciprocal normalization used in this study can be expressed as follows, where the maximum value was considered across all the training samples.

1 $${I}_{D,norm}= \frac{\mathrm{max}(\mathrm{log}{I}_{D})}{\mathrm{log}{I}_{D}},$$

2 $${\mu }_{norm }= \frac{\upmu }{\mathrm{max}(\mu )},$$

3 $${Q}_{N, norm}= \frac{\mathrm{max}(\mathrm{log}{Q}_{N})}{\mathrm{log}{Q}_{N}}.$$

**Figure 1:**
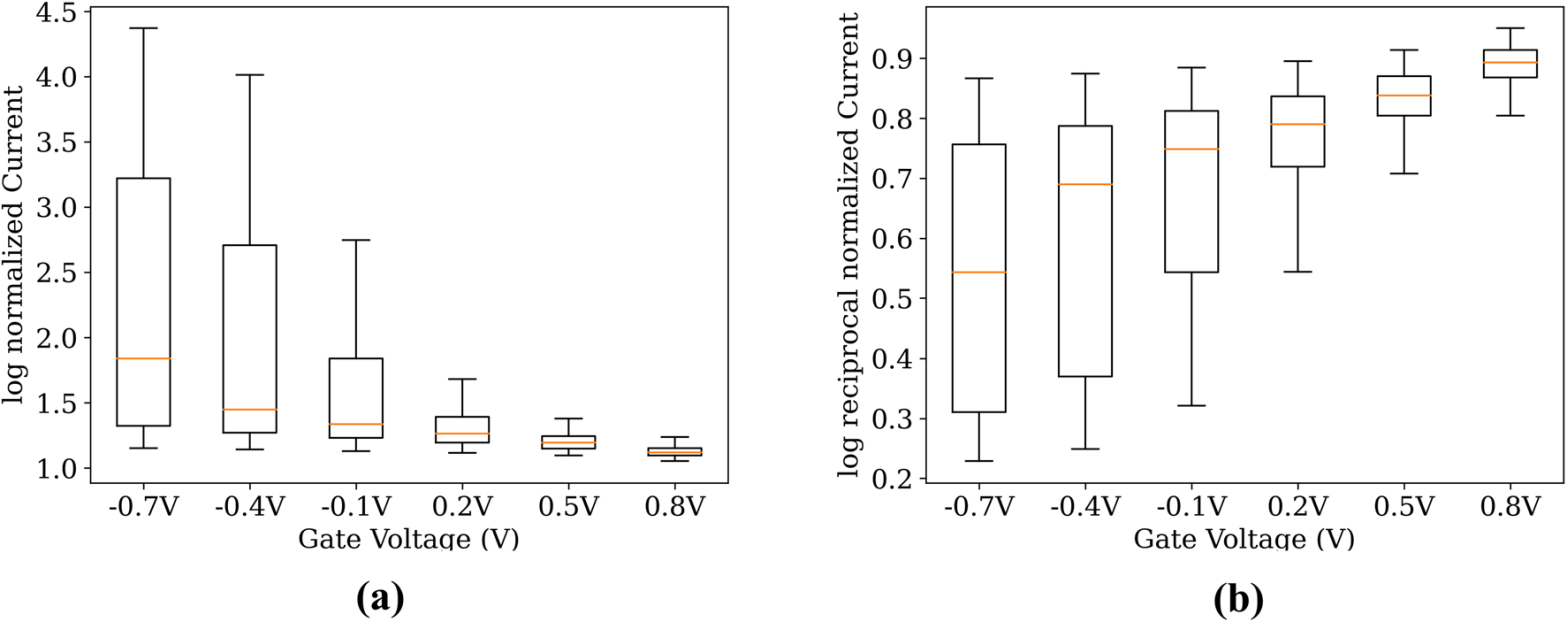
Box plots of normalized ${I}_{D}$ data distributions of 500 validation samples in various voltages were compared using normalization methods. (**a**) data distribution of log-normalized samples; (**b**) data distribution of log-reciprocal normalized samples.

The log-reciprocal normalization was proposed for the semiconductor design problems owing to the special characteristic of the primary properties. In particular, the values of ${I}_{D}$ and ${Q}_{N}$ at the subthreshold voltages, which are typically under 0 V, were relatively small as compared to those around and over the threshold voltages. The conventional log normalization converts the ${I}_{D}$ and ${Q}_{N}$ values at subthreshold voltages to relatively large negative values compared to the values at threshold voltages and over. Owing to this unbalanced value distribution, the conventional log normalization biases the model to fit the subthreshold voltages, which consequently distorts the transfer characteristics (${I}_{D}/{V}_{G}$) curve. In contrast, the log-reciprocal normalization tends to reduce the extreme values of ${I}_{D}$ and ${Q}_{N}$, and subsequently, prevents the value distribution from having a long tail. Moreover, it preserves the desirable shape and tendency of the ${I}_{D}/{V}_{G}$ curve, which are heavily related to the significant specification of the semiconductor device and is a common topic of research. The distributions of ${I}_{D}$ at various voltages are presented in Fig. 1, which depicts the log-reciprocal normalization converting the data distribution as more Gaussian-like.

Consequently, the log-reciprocal normalization delivered superior performance in predicting the secondary properties that are essential device specifications. The models were trained with 3500 training samples and the relative root–mean–square error (RRMSE) was measured over 500 validation samples. As compared to the conventional log normalization, the log-reciprocal normalization displayed improved or at least equivalent prediction errors for all the models. On average, the log-reciprocal normalization method reduced the RRMSE by 28.2%. In particular, the multilayer perceptron (MLP) model in coordination with the proposed log normalization method delivered performance improvements of 57.5%, 83.8%, and 82.2% as compared to the MLP model with the log normalization, respectively. Moreover, the RRMSEs of the MLP model were respectively 68.2%, 38.3%, and 36.4% lesser than those reported by the best machine learning models using log-reciprocal normalization. The performances of the proposed log-reciprocal normalization and the conventional log normalization are comparatively presented in Table 1 for cases involving various baseline machine learning models. The results implied that the log-reciprocal normalization can improve the data presentation for the semiconductor simulation and design problems.

**Table 1:** Performance comparison between log normalization and log reciprocal normalization for the prediction of the secondary properties using various machine learning models.

	Log normalization RRMSE (%)			Log reciprocal normalization RRMSE (%)		
	${S}_{Sw}$	${V}_{Th}$	${\mu }_{Deg}$	${S}_{Sw}$	${V}_{Th}$	${\mu }_{Deg}$
Linear	84.9	16.0	6.5	60.7	15.6	6.5
SVM	106.1	49.6	8.8	70.8	41.8	7.9
Random forest	31.8	15.2	3.3	24.9	14.9	3.3
MLP	18.6	56.7	11.8	**7.9**	**9.2**	**2.1**

Significance values are given in bold.

The RRMSE metric is used for the evaluation.

### Simulator acceleration model

The proposed simulator acceleration model was developed to accelerate the existing FinFET channel simulator while achieving prediction results that are comparable to those obtained using traditional simulators. In general, the traditional simulators can predict the device properties by numerically solving the differential equations that describes the electromagnetic relationships. In contrast, the acceleration models aim to approximate the traditional simulators by finding an implicit function between the properties and design parameters based on the accumulated simulation data. Herein, the proposed acceleration model was designed to predict the three primary properties of the FinFET device (${I}_{D}$, $\mu$, ${Q}_{N}$) based on the four design parameters (${W}_{T}$, ${W}_{B}$ ${T}_{Si}$, ${V}_{Bg}$).

The proposed simulation accelerator aimed to predict both the primary and secondary properties according to the device design. As a traditional simulator can generally predict only the primary properties, the secondary properties are calculated using explicitly defined equations. Similarly, the machine learning model of the accelerator can be trained to target only the primary properties, in which case, the loss function can be expressed as follows.

4 $$Loss =\sum_{i}^{batch}\left\{{\left({{I}_{D,{norm_i}}}-{{\widehat{I}}_{D,{norm_i}}}\right)}^{2}+{\left({{\upmu }_{{norm_i}}}-{{\widehat{\mu }}_{{norm_i}}}\right)}^{2}+ {\left({{Q}_{N,{norm_i}}}-{{\widehat{Q}}_{N,{norm_i}}}\right)}^{2}\right\}.$$

However, in such an approach, small prediction errors in the primary properties can induce large distortions in the calculation of the secondary properties. Thus, a combined loss function considering both the primary and secondary properties was proposed.

5 $${L}_{combined}=\sum_{i}^{batch}\left\{{\left({{I}_{D,{norm_i}}}-{{\widehat{I}}_{D,{norm_i}}}\right)}^{2}+{\left({{\upmu }_{{{norm_i}}}}-{{\widehat{\mu }}_{{{norm_i}}}}\right)}^{2}+ {\left({{Q}_{N,{norm_i}}}-{{\widehat{Q}}_{N,{norm_i}}}\right)}^{2}+ {\left({{S}_{{{Sw_i}}}}-{{\widehat{S}}_{Sw,{loss_i}}}\right)}^{2}+ {\left({{V}_{{{Th_i}}}}-{{\widehat{V}}_{Th,{loss_i}}}\right)}^{2}+ {\left({{\mu }_{{Deg_i}}}-{{\widehat{\mu }}_{Deg,{loss_i}}}\right)}^{2}\right\}.$$

Here, the prediction of the primary properties, ${\widehat{I}}_{D,norm}$, ${\widehat{\mu }}_{norm}$, and ${\widehat{Q}}_{N,norm}$ are the direct output of the acceleration model and the prediction of the secondary properties, ${\widehat{S}}_{Sw}$, ${\widehat{V}}_{Th}$, and ${\widehat{\mu }}_{Deg}$ were estimated from the predicted primary properties using their explicit relations. In the training step, the simulator was trained to approximate the secondary properties, ${\widehat{S}}_{Sw,loss}$, ${\widehat{V}}_{Th,loss}$, and ${\widehat{\mu }}_{Deg,loss}$ with additional layer instead of calculating with explicit relation equations since the equations that derives the secondary properties are not differentiable. However, in the validation and verification step of the model, the secondary properties, ${\widehat{S}}_{Sw}$, ${\widehat{V}}_{Th}$, and ${\widehat{\mu }}_{Deg}$ estimated from the predicted primary properties were used.

As described in the Methods section, a two-layered MLP model was developed and trained with 3500 training samples using the proposed combined loss function. Additionally, the parameters were tuned with 500 validation samples, and the effectiveness of the proposed combined loss function was verified by comparing the performance of the proposed model with that of several machine learning methods trained only using the primary property losses. Subsequently, the performances were evaluated by comparing the RRMSEs of the ${\widehat{S}}_{Sw}$, ${\widehat{V}}_{Th}$, and ${\widehat{\mu }}_{Deg}$ observed in the prediction results. Overall, the performances were measured over 500 validation and 1000 test samples.

**Figure 2:**
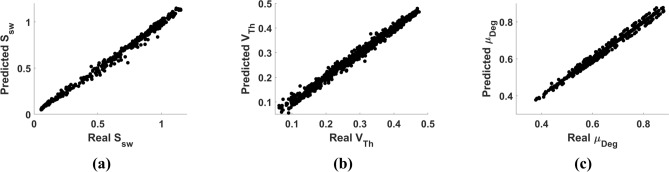
Scatter plots of predicted and true secondary property: (**a**) ${S}_{Sw}$, (**b**) ${V}_{Th}$, and (**c**) ${\mu }_{Deg}$ of 1000 test samples evaluated using primary properties predicted with proposed simulator acceleration model.

The proposed simulator acceleration model with the combined loss function displayed high accuracy in predicting both the primary and secondary properties. In the 1000 test samples, the RRMSEs of the primary properties ${\widehat{I}}_{D,norm}$, ${\widehat{\mu }}_{norm}$, and ${\widehat{Q}}_{N,norm}$ were 0.0028%, 0.0020%, and 0.0022%, and those of the secondary properties ${\widehat{S}}_{Sw}$, ${\widehat{V}}_{Th}$, and ${\widehat{\mu }}_{Deg}$ were 5.7%, 3.6%, and 1.3%, respectively. These improvements are noteworthy in comparison to the baseline models. Moreover, the RRMSEs of the proposed combined-loss MLP in the 1000 test samples were 30.5%, 69.7%, and 48.0% lesser than those of the best alternative methods, respectively. Additionally, all the R^2^ scores of the secondary properties determined using the combined loss MLP model were beyond 0.99. The results are comparatively presented in Table 2. Furthermore, the scatter plots between the predicted and real secondary property values are presented in Fig. 2, which demonstrates good agreement of the prediction results. Thus, the results implied that the combined-loss MLP model can successfully learn the tendency of the primary properties and preserve the shape of the primary property curves as compared to the alternative baseline models.

**Table 2:** Performance comparison of the proposed combined loss MLP model with other machine learning models for secondary property predictions.

	Validation set RRMSE (%)			Test set RRMSE (%)		
	${S}_{Sw}$	${V}_{Th}$	${\mu }_{Deg}$	${S}_{Sw}$	${V}_{Th}$	${\mu }_{Deg}$
Linear	60.5	14.2	5.9	68.9	14.7	6.3
SVM	74.5	41.5	7.8	80.0	39.9	8.0
Random forest	32.9	23.7	4.9	37.2	24.2	5.2
MLP	8.8	11.7	2.4	8.2	11.9	2.5
Combined loss MLP	5.3	6.7	1.8	5.7	3.6	1.3

The RRMSE metric is used for the evaluation.

More importantly, the proposed simulator acceleration model successfully reduced the computation time compared to the traditional FinFET simulator. The average computational time of a FinFET simulator is 70 s/sample and a total of 90 h is required to simulate 5000 samples. Comparatively, the proposed simulator acceleration model required only 2.52 s to calculate 5000 samples, which is approximately 122,000 times faster than the traditional FinFET simulator.

### Inverse-design model

In addition to the development of the specialized preprocessing method and loss function for the semiconductor design problems, we demonstrate the utility of the deep neural networks for the semiconductor inverse-design problem. The inverse-design model aimed to directly predict the design of a semiconductor device that holds same specifications with the desired specifications which are the input of the model. Thus, the proposed inverse-design model aimed to predict the four design parameters of the FinFET device (${W}_{T}$, ${W}_{B}$ ${T}_{Si}$, ${V}_{Bg}$) from the desired secondary properties of the FinFET device (${S}_{Sw}$, ${V}_{Th}$, ${\mu }_{Deg}$). However, the inverse-design problems generally have multiple solutions, implying that specifications with marginal difference can be presented from distinct designs. To handle the limitation that the problem is not an injective function, implementing fine-tuned the deep neural network enables the model to converge into one of the possible designs rather than to converge into the average of all of the possible designs. Hence, implementing a deep neural network is also feasible for solving the inverse-design problem.

Similar to the simulator acceleration model, a two-layered MLP model was developed and trained with 3500 training samples. In addition, the parameters were tuned with 500 validation samples, and the performance of the model was evaluated by comparing the desired specifications used as an input of the model with the actual specifications of the designed device, which were derived from the traditional FinFET simulator. Subsequently, the performance was measured over 1000 random specifications.

**Figure 3:**
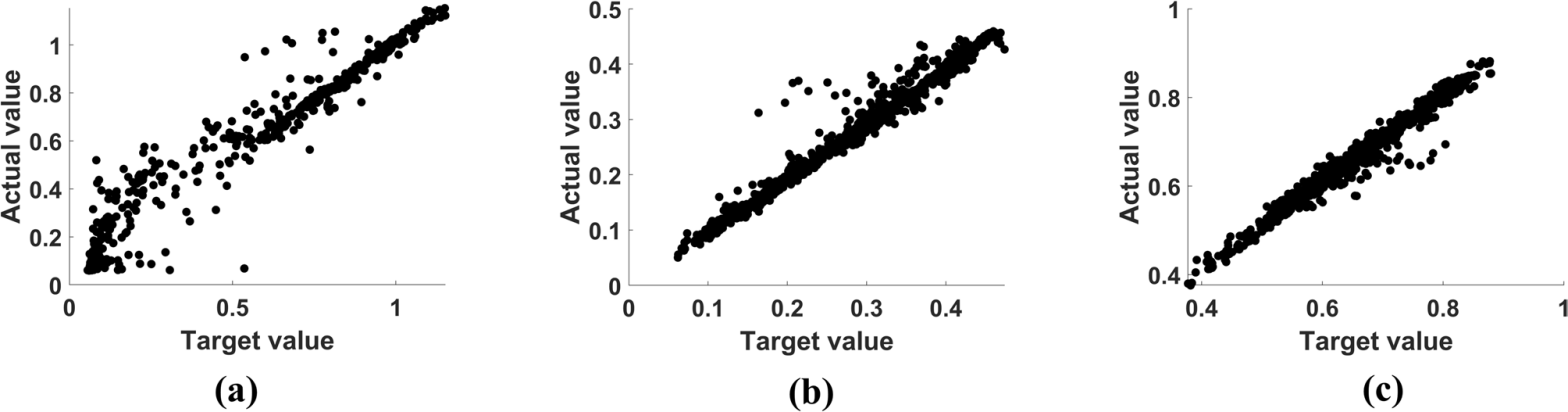
Scatter plots of desired target and actual specifications of secondary property: (**a**) ${S}_{Sw}$, (**b**) ${V}_{Th}$, and (**c**) ${\mu }_{Deg}$.

Upon evaluating with the actual specifications, the proposed model displayed adequate performance in design prediction. In particular, the performance of the inverse design was evaluated based on the error between the target and actual specifications of the predicted designs as calculated using the FinFET simulator. As depicted in Fig. 3, the target and actual specifications agreed well with each other for the all the three secondary properties (${S}_{Sw}$, ${V}_{Th}$, ${\mu }_{Deg}$). Moreover, the R^2^ scores of ${S}_{Sw}$, ${V}_{Th}$, and ${\mu }_{Deg}$ were measured as 0.96, 0.97, and 0.97, respectively. Although the proposed design prediction appropriately satisfied the desired specifications, yielding the desired ${S}_{Sw}$ is challenging, especially for low values. This is probably because ${S}_{Sw}$ is a kind of gradient that is easily distorted with small errors. In contrast, the actual ${V}_{Th}$ and ${\mu }_{Deg}$ of 1000 cases calculated from the predicted designs were highly similar to the target ${V}_{Th}$ and ${\mu }_{Deg}$. In detail, the variations in 91% and 99% of ${V}_{Th}$ and ${\mu }_{Deg}$ were within 10% of the target specifications, whereas those in 78% of ${S}_{Sw}$ were under 10%. Upon considering all the three specifications, 73% of cases were determined within the 10% range of the target specifications. The detailed comparison results of the actual and target specifications in terms of tolerance are presented in Table 3.

**Table 3:** Percentages of the design cases that meet the target specifications within specific relative tolerance.

Tolerance (%)	${S}_{Sw}$ (%)	${V}_{Th}$ (%)	${\mu }_{Deg}$ (%)	All (%)
< 20	81.9	98.7	99.9	80.9
< 10	78.2	91.0	98.9	72.5
< 5	72.9	66.6	93.1	51.1

The tolerance is the allowed maximum ratio of the difference between the actual and target values over the target value.

Overall, the predicted design parameters displayed positive correlations with the original design parameters used in generating the test samples (Fig. S1), which implied that the proposed design prediction followed the general trends. The R^2^ values of ${W}_{T}$, ${W}_{B}$, ${T}_{Si}$, and ${V}_{Bg}$ were 0.47, 0.69, 0.75, and 0.96, respectively. The proposed design prediction reproduced the original design parameters of ${V}_{Bg}$ almost identically, whereas the remaining three design parameters (${W}_{T}$, ${W}_{B}$, and ${T}_{Si}$) exhibited relatively less correlation.

**Figure 4:**
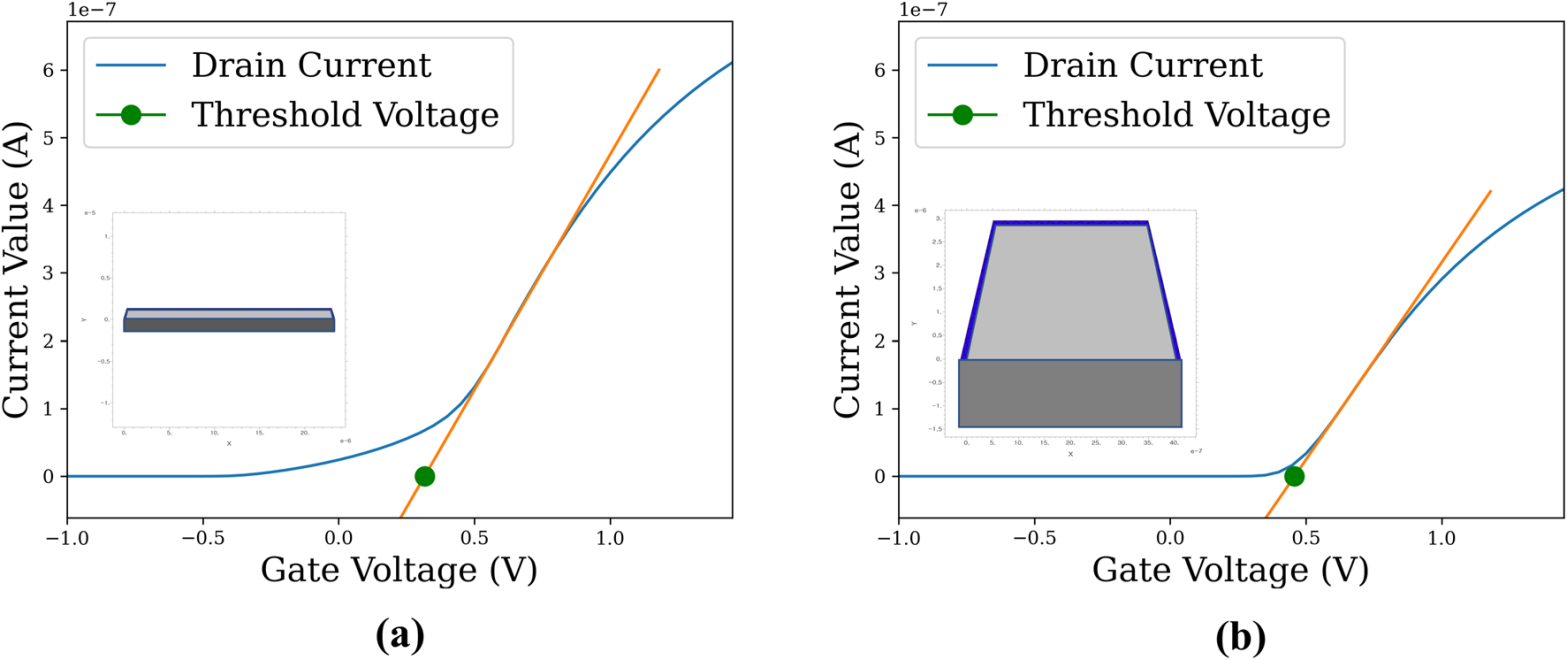
The ${I}_{D}/{V}_{G}$ graphs of an exemplary case of inverse-design problem. (**a**) ${I}_{D}/{V}_{G}$ curve of test sample with target specification; (**b**) ${I}_{D}/{V}_{G}$ curve of predicted design evaluated using inverse-design model.

Notably, the design prediction results clearly demonstrate that the inverse-design problem holds multiple feasible solutions. These result supports the reasonability of verifying the proposed model with the specifications calculated from the predicted design rather than verifying with the design prediction results. The desired specifications were well satisfied, regardless of certain discrepancies in the predicted and original design parameters. A typical example is presented in Fig. 4, which demonstrates the ${I}_{D}/{V}_{G}$ curve and the channel design of the two distinct samples. The blue lines in Fig. 4 denote the ${I}_{D}/{V}_{G}$ curves calculated using the FinFET simulator based on the design parameters, and the channel design of the samples is located above the ${I}_{D}/{V}_{G}$ curve. Additionally, the orange lines in Fig. 4 express the linear gradients extracted from the amplifying region of the ${I}_{D}/{V}_{G}$ curve. Moreover, the green dots in Fig. 4 denote the threshold voltage (${V}_{Th}$) of the samples derived from the orange lines. Figure 4a depicts the results of a sample selected from the test set with relatively low ${T}_{Si}$ as compared to ${W}_{T}$ and ${W}_{B}$. Comparatively, Fig. 4b depicts the results of a sample predicted using the inverse-design model to have the same specifications as that in Fig. 4a. Although the design results presented in Fig. 4a,b are remarkably distinct, the shapes of the ${I}_{D}/{V}_{G}$ curves correlated to the threshold voltage of the device are similar. Thus, the proposed design prediction truly learned the relationship between the FinFET design and its specification rather than remembering the training samples. These results further portray the potential of the proposed method to contribute toward extending human design scopes.

## Conclusion

Overall, the results demonstrated that the proposed deep neural network models can be used to resolve both the simulation acceleration and inverse-design problems for FinFET devices. In particular, the log-reciprocal normalization in the data preprocessing step improved the prediction accuracy during simulation by balancing the severely skewed data distribution. In addition, the proposed combined loss in the simulation acceleration model enabled the accurate prediction of primary properties as well as the further calculation of secondary properties. As compared to the traditional simulators, the proposed simulation acceleration model achieved comparable prediction results (R^2^ = 0.99) and was more than 120,000 times faster. Moreover, the deep learning model can be applied to directly predict the design parameters using the desired specifications as a typical inverse-design problem. The proposed deep learning-based solution for the inverse-design problem which aims to find one feasible design solution among possible multiple solution to assist the design process have shown the novel performance. The actual design specifications derived by the prediction results of the proposed model corresponded well with the originally desired specifications. Interestingly, we also have identified cases with two distinct designs for the same specifications, which implied that the proposed model actually learns the design-specification relations, not just remembering the train cases. Overall, 73% of the designed cases obtained using the proposed model satisfied the desired specification of all the three secondary properties with a 10% error tolerance, which provides a good starting point for a human expert to initiate the design process.

Additionally, our proposed method showed clear advantage compared to existing semi-conductor inverse-design solutions^12^. Compared to the existing method that implements an evolutionary algorithm which indirectly predicts the appropriate design parameters of the desired component through the trial and errors, our proposed method directly predicts the design parameters of the desired specifications. This direct prediction guarantees the convergence of the design and saves the computational cost compared to the evolutionary algorithm since the convergence of indirect prediction is not guaranteed.

Although this study focused on a relatively simple FinFET device, a similar approach can be applied to more complex devices or a small circuit of several FinFET devices. In general, the discussed machine learning and deep learning approaches will aid in resolving several device and circuit-related problems that can be further extended to a general simulation acceleration and inverse-design problems.

## Data and methods

### Data generation

**Figure 5:**
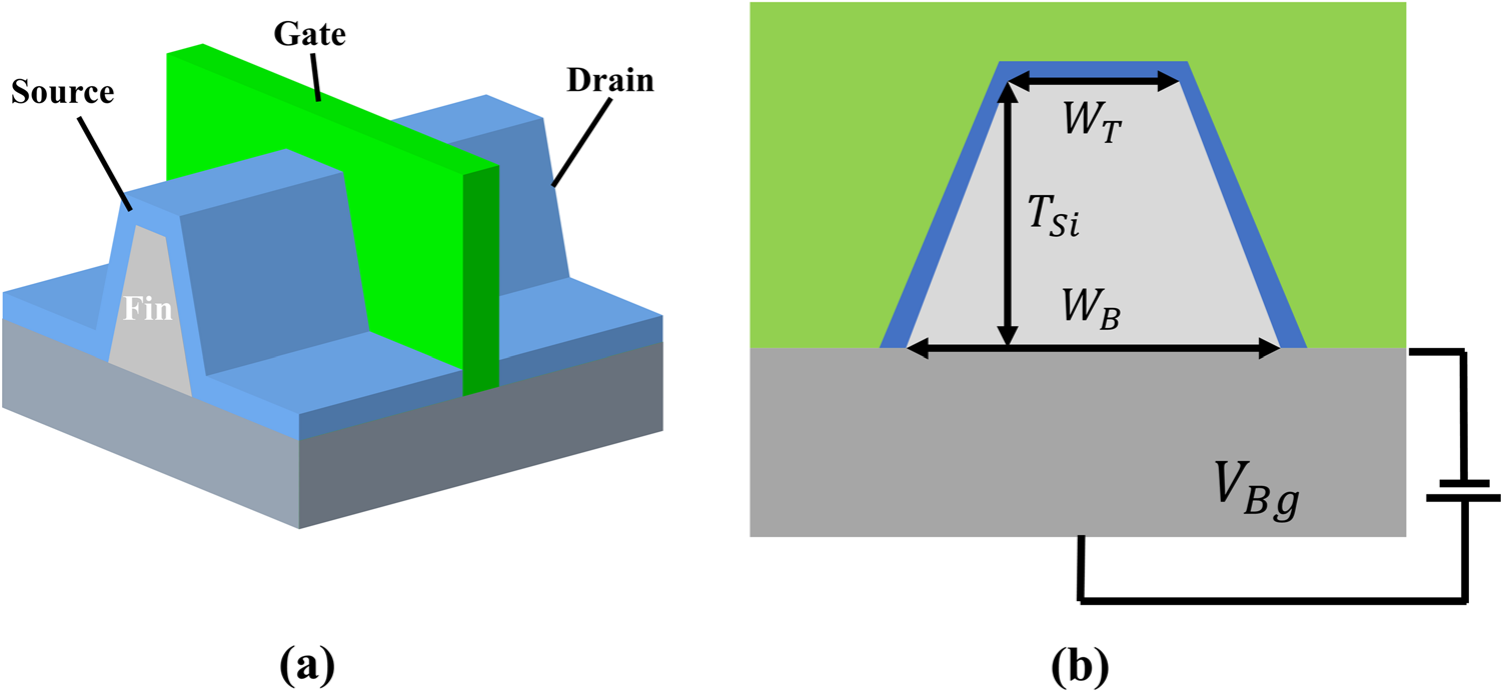
(**a**) 3D outline schematic of FinFET device; (**b**) 2D cross-sectional schematic of channel of FinFET device channel. Green, blue, light gray, and dark gray regions represent the gate, silicon oxide, silicon channel, and oxide box of the device, respectively. The figures were drawn with the Creo 7 educational version provided by PTC (www.ptc.com).

A traditional simulator was constructed to characterize the device parameter of a given FinFET device design using commercial software FlexPDE (www.pdesolutions.com). The general 3D schematic and 2D cross-section of a FinFET device are presented in Fig. 5, wherein Fig. 5a displays the cross-section of the gate component of the gray fin channel. In addition, the blue, green, and gray regions in Fig. 5b correspond to the oxide, silicon, and oxide box of the channel, respectively. The traditional simulator solved the Maxwell equations using the boundary conditions derived from a given design to characterize the device parameter. The detailed equation derived from the Gauss’s Law is expressed as follows.

6 $$\nabla ^\circ \left(\varepsilon \nabla {V}_{G}\right)= {Q}_{Total}.$$

The above equation denotes that the total charge in a given region ($${Q}_{Total})$$ is equal to the divergence ($$\nabla ^\circ )$$ of electromagnetic permittivity ($$\varepsilon$$) multiplied with the gradient of gate voltage ($$\nabla {V}_{G})$$. In particular, the FlexPDE was used to numerically solve the derived partial differential equations. The detailed conditions of this simulator are listed in Table S2.

As depicted in Fig. 5b, a FinFET structure is mainly specified according to the four design parameters, which include the top and bottom width of the FinFET channel $({W}_{T}$, ${W}_{B}$), the channel thickness (${T}_{Si}$), and the box gate voltage (${V}_{Bg}$) imposed on the silicon box. In addition, the ${W}_{T}$, ${W}_{B}$, and ${T}_{Si}$ determine the silicon/oxide border length, which is considered as an effective channel dimension parameter that significantly influences the device parameter of a FinFET device. Moreover, the ${V}_{Bg}$ imposed on the box under the silicon channel influenced the overall conductivity across all voltages. Thus, the experiments were focused on these four parameters as these parameters are significantly correlated with the device parameter of the semiconductor device and can be conveniently controlled during the manufacturing process.

Based on the design specified by the four parameters, the primary device parameter were evaluated according to the gate voltage (${V}_{G}$) variations by solving the differential equations using the traditional simulator. In particular, the variations in the drain current flow (${I}_{D}$), effective mobility ($\mu$), and electron charge density (${Q}_{N}$) with respect to the varying gate voltage (${V}_{G}$) were calculated using the following equations.

7 $${I}_{D}= \sigma {V}_{D},$$

8 $$\mu = \frac{\sigma }{{Q}_{n}L},$$

9 $${Q}_{n}=q\int {{n}_{i}e}^{\frac{{V}_{G}}{kT}},$$

10 $$\sigma = \oint \frac{{\mu}q{n}_{i}}{L}.$$

The ${I}_{D}$, $\mu$, and ${Q}_{N}$ values of the device were calculated using the simulator at 50 distinct ${V}_{G}$ values. The range of ${V}_{G}$ was set from − 1 to 1.45 V with an interval of 0.05 V per observation.

Moreover, the three secondary properties of a FinFET device were derived directly from the primary properties, which are essential for the actual utilization of a semiconductor device^13^. In particular, the subthreshold swing (${S}_{Sw}$), threshold voltage (${V}_{Th}$), and mobility degradation (${\mu }_{Deg}$) were derived as the secondary properties of the FinFET device, which were characterized by the following equations.

11 $${S}_{Sw}=min\left\{\frac{1}{\frac{d}{d{V}_{G}}\mathrm{log}{I}_{D}}\right\},$$

12 $${V}_{Th}= \frac{{I}_{d,{V}_{a}}- \frac{d{I}_{d,{V}_{a}}}{d{V}_{G}} {V}_{a}}{\frac{d{I}_{d,{V}_{a}}}{d{V}_{G}}},$$

13 $${\mu }_{Deg} = \frac{{\mu }_{1.0}}{{\mu }_{ max}}.$$

A subthreshold swing corresponds to the minimum value of the reciprocal of a gradient of the log ${I}_{D}$ in terms of ${V}_{G}$. A threshold voltage represents the turn-on voltage of a semiconductor. The ${V}_{a}$ denotes the gate voltages with the ${I}_{d}/{V}_{g}$ graph present in the linear region, and the ${I}_{d,{V}_{a}}$ denotes the value of drain current at the gate voltage ${V}_{a}$. Moreover, the mobility degradation represents a ratio between the effective mobility value at a certain voltage and the maximum effective mobility value. In particular, ${\mu }_{1.0}$ denotes the effective mobility value at a gate voltage that is 1 V higher than the threshold voltage of the given semiconductor, and ${\mu }_{max}$ represents the maximum effective mobility value of a given semiconductor.

Subsequently, the trapezoid-shaped channel FinFET device samples were generated by modifying the four design parameters (${W}_{T}$, ${W}_{B}$, ${T}_{Si}$, ${V}_{Bg}$) using the traditional simulator. The ranges of these design parameters were defined in physically reasonable regions that can be applied in actual manufacturing processes. In particular, the bottom width of the sample was randomly selected within 10–250 nm, whereas the top width of the sample was randomly selected in between 1 nm and the bottom width. Additionally, the silicon thickness of the sample was randomly selected between 10 and 50 nm. Lastly, the box gate voltage of the sample was randomly selected between 0 and 40 V.

A total 5000 samples were generated using the traditional simulator, and among these 5000 samples, 3500 samples were randomly selected as the training samples, 500 samples were randomly selected as the validation samples, and 1000 samples are randomly selected as the test samples. The RRMSE was calculated using the following equation and used as an evaluation metric of the proposed models in the validation and test stages.

14 $$\mathrm{RRMSE }(\mathrm{\%}) = \frac{\sqrt{\frac{1}{n} {\sum }_{i-1}^{n}{({y}_{i}^{real}- {y}_{i}^{predict})}^{2}}}{\frac{1}{n}{\sum }_{i=1}^{n}{y}_{i}^{real}} \times 100.$$

### Model training

The simulator acceleration model was constructed based on the training samples generated from the traditional simulator to predict the three primary device parameter (${I}_{D}$, $\mu$, ${Q}_{N}$) of the FinFET device using the four design parameters (${W}_{T}$, ${W}_{B}$ ${T}_{Si}$, ${V}_{Bg}$). Similar to the traditional simulator, the proposed acceleration model predicted a total of 150 distinct values of the (${I}_{D}$, $\mu$, ${Q}_{N}$) ranging from − 1 to 1.45 V in 0.05 V interval of ${V}_{G}$. The proposed simulator acceleration model is an MLP model integrated with a specialized combined loss function, as discussed earlier. In addition, the proposed model comprises two hidden layers—each containing 128 nodes with a rectified linear unit (RELU) operating as an activation function for the hidden layers and a sigmoid acting as an activation function for the output layer. During the training procedure, the proposed model was trained for 1000 epochs with a batch size of 256. Moreover, an adaptive moment estimation (ADAM) optimizer was implemented with an initial learning rate of 0.01 with 0.99 decay for every 75 steps to train the model. Furthermore, the proposed model was trained with a NVDIA RTX 2080 SUPER GPU and an INTEL 4-core CPU i7-7700k.

An MLP structure was proposed for the inverse-design model as well. The inverse-design model was developed to predict the four design parameters (${W}_{T}$, ${W}_{B}$ ${T}_{Si}$, ${V}_{Bg}$) of the FinFET device by utilizing the target specifications, i.e., the three secondary properties (${S}_{Sw}$, ${V}_{Th}$, ${\mu }_{Deg}$). In particular, the MLP model of the inverse-design problem comprises two hidden layers—the first layer with 256 nodes and the second layer with 32 nodes. Moreover, the RELU activation was used for all the hidden layers, and the sigmoid function was used as an activation function for the output layer. During the training procedure, the proposed model was trained with a batch size of 32 for 300 epochs, and an ADAM optimizer was implemented as an optimizer to train the model at a learning rate of 0.003. The proposed inverse-design model was trained with a NVDIA RTX 2080 SUPER GPU and an INTEL 4-core CPU i7-7700k.

## Supplementary Information

Supplementary Information.
