# Supplementary material for: Simulator acceleration and inverse design of fin field-effect transistors using machine learning
Source: Sci Rep. 2022 Jan 21;12:1140. doi: 10.1038/s41598-022-05111-3 (PMC8782883; doi:10.1038/s41598-022-05111-3)
Supplement: Supplementary file 1 — Supplementary Information. [file 41598_2022_5111_MOESM1_ESM.docx]

**Supplementary Materials**

**Simulator Acceleration and Inverse Design of Fin Field-Effect Transistor using Machine Learning**

Insoo Kim^1^, So Jeong Park^1^, Changwook Jeong^2^, Munbo Shim^2^, Dae Sin Kim^2^, Gyu-Tae Kim^1^, Junhee Seok^1,*^

^1^School of Electrical Engineering, Korea University, Seoul, Korea

^2^Computational Science and Engineering Team,

Data and Information Technology Center, Samsung Electronics, Samsungjeonja-ro, Hwaseong-si,Gyeonggi-do 18448, South Korea

* Correspondence should be addressed to jseok14@korea.ac.kr

Table S1. Detailed description of design parameters, primary electromagnetic properties, and secondary electromagnetic properties of a FinFET device used in the experiments.

| **Variable** | **Description** | **Value type** |
| --- | --- | --- |
| $W_{T}$ | FinFET channel top width | Design parameter |
| $W_{B}$ | FinFET channel bottom width | Design parameter |
| $T_{Si}$ | FinFET channel thickness | Design parameter |
| $V_{Bg}$ | Back gate Voltage | Design parameter |
| $I_{D}$ | Drain Current | Primary property |
| $\mu$ | Effective mobility | Primary property |
| $Q_{N}$ | Electron charge density | Primary property |
| $S_{Sw}$ | Minimum value of gradient of log $I_{D}$ versus $V_{G}$ | Secondary property |
| $V_{Th}$ | Voltage where $I_{D}$and $V_{G}$ starts to show linear relation. | Secondary property |
| $\mu_{Deg}$ | The ratio of effective mobility due to the maximum effective mobility at specific voltage | Secondary property |

Table S2. Detailed description of the conditions of the FinFET simulator that generated the sample used throughout the experiments.

| **Type** | **Equation** | **description** |
| --- | --- | --- |
| Variable | V | effective voltage |
| Design | W_top = 1~W_bot | Channel top width ($W_{T}$), nm |
| Design | W_bot = 10 ~ 250 | Channel bottom width ($W_{B}$), nm |
| Design | tsi = 10~50 | Channel thickness ($T_{Si}$), nm |
| Constant | tox = 1.2 | EOT-Gate oxide thickness, nm |
| Constant | tbox=145 | Real box thickness, nm |
| Constant | ratio = 10 | Ratio of the effective box |
| Constant | tboxeff = tbox/ratio | Unit, nm |
| Constant | L = 1000 | Device Length, nm |
| Constant | k = 86e-6 | k/q, re-Boltzman constant, 8.617e-5 V/K |
| Constant | T = 300 | Temperature, K |
| Constant | q = 1.6e-19 | Elementary charge, C |
| Constant | Nd = 1.4e-11 | Si doping, /nm^3^ |
| Constant | ni = 1.4e-11 | Intrinsic doping, /nm^3^ |
| Constant | Ng = ni | TiN gate doping, midgap material |
| Constant | Vfb=-k*T*ln(ni^2^/Ng/Nd) | Flat band voltage |
| Constant | epssi = 12*8.85e-21 | Si permittivity, F/nm |
| Constant | epsox = 4*8.85e-21 | SiO_2_ permittivity, F/nm |
| Constant | epsbox = epsox/ratio | Thickness compensation |
| Constant | eps0 = 8.85e-21 | Vacuum permittivity, F/nm |
| Equation | Cox=epsox/tox*(W_top+2*((((W_bot-W_top)*0.5)^2^+tsi^2^)^0.5^)) | 2D-GateOxide capacitance |
| Constant | mu0 = 400 | Max mobility |
| Constant | n0 = Nd | Electron concentration |
| Constant | p0 = ni^2^/Nd | Hole concentration |
| Equation | E = -$\nabla$V | Electric field in x axis |
| Equation | Emag = \|E\| | Magnitude of electric field |
| Constant | Ec = 0.1 | Critical electric field in high-doped Si, V/nm |
| Constant | alpha = 1.5 | Power law for mobility attenuation |
| Variable | Vg = 2.5*(stage-1)/50 -1 | Gate Voltage, -1~1.45V |
| Constant | Vd = 0.01 | Effective drain Voltage, V |
| Design | Vbox = 0~40 | Back gate Voltage, V |
| Equation | $mu$=mu0/(1+(Emag/Ec)^alpha^) | Mobility with degradation factor |
| Constant | n = n0*exp(V/kT) | Electron concentration in a field |
| Constant | p = p0*exp(V/kT) | Hole concentration in a field |
| Output | Qn = q$\int(n,2)$ | Electron charge density ($Q_{N}$) |
| Equation | Gd = $∯ (mu$*q*n,2) | Conductance |
| Output | Id = Gd * Vd | Drain current ($I_{d}$), V |
| Output | Mueff = Gd/(Qn*L) | Effective mobility ($\mu$) |
| Equation | V = Vg – Vfb | Line voltage, V |
| Equation | $\nabla^{\circ} \left( \varepsilon\nabla V_{G} \right)$ = 0 | Gauss Law |


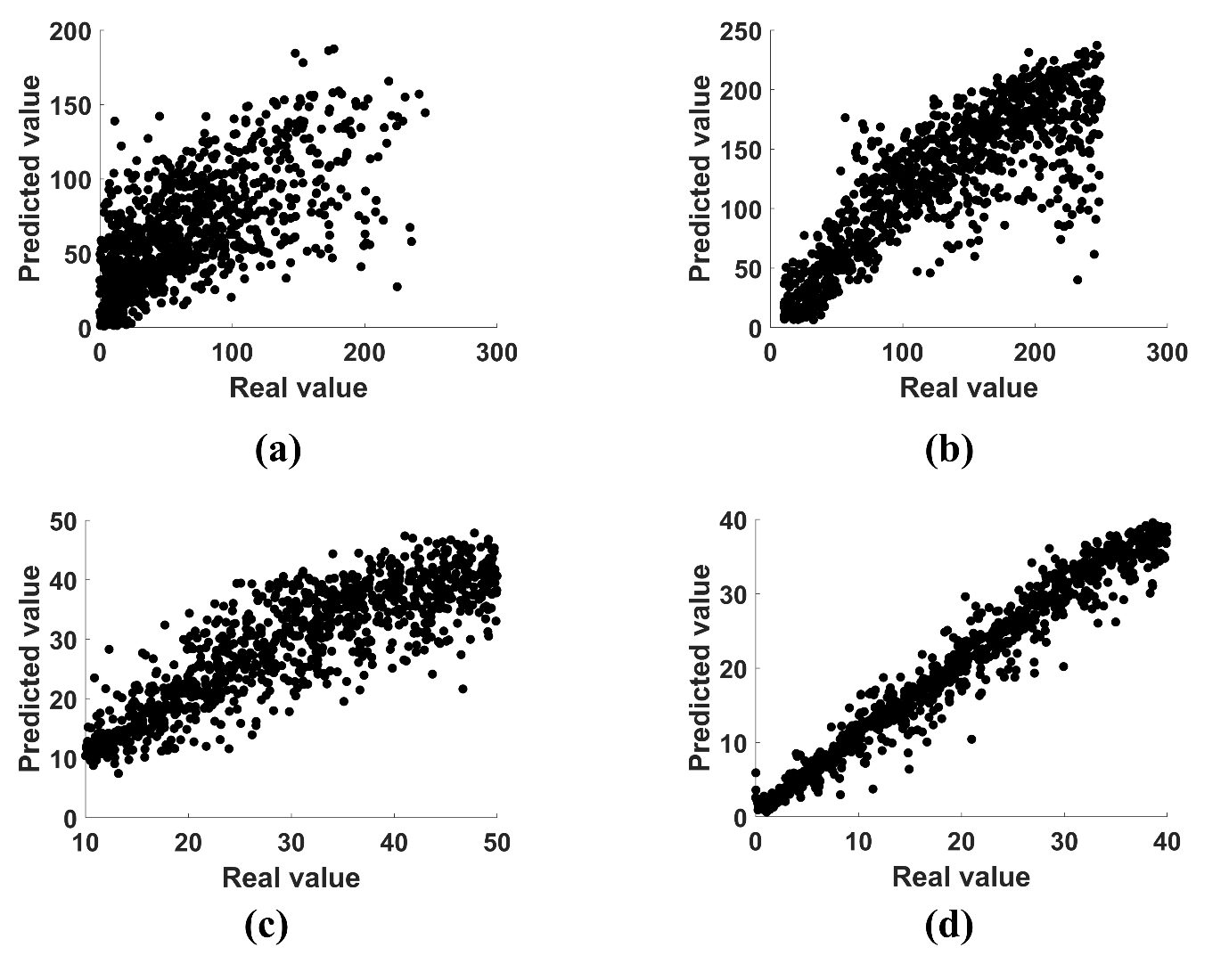


Figure S1. Scatter plots of the semiconductor device designs (a) $W_{T}$, (b) $W_{B}$, (c) $T_{Si}$, and (d) $V_{Bg}$ of 1,000 test samples and predicted designs which are the output values of the inverse design model.
